# Enabling HighThroughput Electron Cryo-microscopy for Drug Discovery

**DOI:** 10.1063/4.0000881

**Published:** 2025-10-27

**Authors:** Pamela A Williams

**Affiliations:** Astex Pharmaceuticals, Cambridge, Cambs, United Kingdom

## Abstract

Structure and fragment based drug discovery campaigns (SBDD and FBDD) rely on frequent access to high resolution protein-ligand structures, in order to support the design-make-test-analyse cycle. Protein crystallography is widely used to support such medicinal chemistry campaigns, but in recent years electron cryo-microscopy (cryo-EM) has emerged as alternative approach that may be applied to proteins that are recalcitrant to crystallisation. In this talk I will describe the advances that have enabled this, provide an illustration of how Astex uses cryo-EM to support drug development and reflect upon the additional developments that will be required if cryo-EM is to reach its full potential in both SBDD and FBDD.

